# The deubiquitinase USP11 is a versatile and conserved regulator of autophagy

**DOI:** 10.1016/j.jbc.2021.101263

**Published:** 2021-09-30

**Authors:** Mila Basic, Alexandra Hertel, Justyna Bajdzienko, Florian Bonn, Mariana Tellechea, Alexandra Stolz, Andreas Kern, Christian Behl, Anja Bremm

**Affiliations:** 1 Institute of Biochemistry II, Goethe University Frankfurt - Medical Faculty, University Hospital, Frankfurt am Main, Germany; 2 Buchmann Institute for Molecular Life Sciences, Goethe University Frankfurt, Frankfurt am Main, Germany; 3 Institute of Pathobiochemistry, University Medical Center of the Johannes Gutenberg University, Mainz, Germany

**Keywords:** autophagy, proteostasis, ubiquitin, deubiquitinase (DUB), PI3KC3-C1, mTORC1, Aβ, amyloid-β, ACN, acetonitrile, BSA, bovine serum albumin, DUB, deubiquitinase, hAβ42, human amyloid-β protein 1 to 42, IP, immunoprecipitation, LFQ, label-free quantification, mTORC1, mechanistic target of rapamycin complex 1, NHT, nonhuman targeting, NRBF2, nuclear receptor-binding factor 2, PI(3)P, phosphatidylinositol-3-phosphate, PI3KC3-C1, class III phosphatidylinositol 3-kinase complex I, S6K, S6 kinase, ULK1, unc-51-like autophagy activating kinase 1, USP11, ubiquitin-specific protease 11, WIPI, WD-repeat domain phosphoinositide-interacting protein

## Abstract

Autophagy is a major cellular quality control system responsible for the degradation of proteins and organelles in response to stress and damage to maintain homeostasis. Ubiquitination of autophagy-related proteins or regulatory components is important for the precise control of autophagy pathways. Here, we show that the deubiquitinase ubiquitin-specific protease 11 (USP11) restricts autophagy and that KO of USP11 in mammalian cells results in elevated autophagic flux. We also demonstrate that depletion of the USP11 homolog H34C03.2 in *Caenorhabditis elegans* triggers hyperactivation of autophagy and protects the animals against human amyloid-β peptide 42 aggregation-induced paralysis. USP11 coprecipitated with autophagy-specific class III phosphatidylinositol 3-kinase complex I and limited its interaction with nuclear receptor-binding factor 2, thus decreasing lipid kinase activity of class III phosphatidylinositol 3-kinase complex I and subsequent recruitment of effectors such as WD-repeat domain phosphoinositide-interacting proteins to the autophagosomal membrane. Accordingly, more WD-repeat domain phosphoinositide-interacting protein 2 puncta accumulated in USP11 KO cells. In addition, USP11 interacts with and stabilizes the serine/threonine kinase mechanistic target of rapamycin, thereby further contributing to the regulation of autophagy induction. Taken together, our data suggested that USP11 impinges on the autophagy pathway at multiple sites and that inhibiting USP11 alleviates symptoms of proteotoxicity, which is a major hallmark of neurodegenerative diseases.

Autophagy is a highly conserved catabolic pathway and quality control process that maintains cellular homeostasis by transporting intracellular components to the lysosome for their degradation. Under physiological conditions, autophagy degrades long-lived proteins or damaged organelles. Moreover, autophagy can be induced by environmental stimuli such as starvation, reactive oxygen species, or hypoxia, to generate building blocks for de novo biomolecule synthesis or substrates for energy production (1).

Autophagy is critical for various biological processes, and it has been established as a protective factor against neurodegeneration associated with intracytoplasmic aggregate-prone protein accumulation (2). In addition to neurodegenerative diseases, deregulation of autophagy has been implicated in the progression of cancer, immune diseases, and metabolic disorders. Interestingly, depending on the pathological condition and tissue context, both autophagy induction and inhibition have been considered as therapeutic approaches (3). Therefore, investigating the regulatory mechanisms of autophagy to unveil novel therapeutic strategies has been an ongoing mission in the last years.

Posttranslational modifications, among them most notably phosphorylation and ubiquitination, play an important role in the initiation and regulation of autophagy. The critical role of ubiquitination, the process by which the small protein modifier ubiquitin is attached to substrate proteins by the sequential activities of E1 (ubiquitin-activating), E2 (ubiquitin-conjugating), and E3 (ubiquitin ligase) enzymes, has been demonstrated along multiple steps of the autophagy pathway (4). This ranges from the recognition of cargo to the nucleation of the double membrane and fusion with the lysosome. Ubiquitination also facilitates selected recruitment of autophagy receptors, which act as bridging molecules between the ubiquitinated cargo and autophagosome (5). In addition, various E3 ubiquitin ligases have been shown to impact degradation or stabilization of upstream regulators and components of the autophagy pathway (6).

Ubiquitin signals are regulated and terminated by deubiquitinases (DUBs), of which there are approximately 100 encoded in the human genome (7). DUBs display specificity for both substrates and particular ubiquitin chain types. Active DUBs are necessary to maintain free ubiquitin levels in the cell, rescue proteins from ubiquitin-mediated degradation, and control the dynamics of ubiquitin-mediated signaling events. While multiple DUBs have already been implicated in regulating autophagic substrate degradation (8), our understanding of their detailed action and implications for potential therapeutic strategies is only beginning to emerge.

Mechanistic target of rapamycin complex 1 (mTORC1) is a master regulator of cell growth and metabolism, and its inactivation in response to amino acid starvation is a major trigger for autophagy (9). Relieving of mTORC1-mediated repression of the unc-51-like autophagy-activating kinase 1(ULK1) complex and class III phosphatidylinositol 3-kinase complex I (PI3KC3-C1) is critical for phagophore nucleation (1, 10, 11). PI3KC3-C1 phosphorylates the lipid head group of phosphatidylinositol to generate phosphatidylinositol-3-phosphate (PI(3)P). PI(3)P production is an essential early event in autophagy initiation, occurring just downstream of ULK1, and serves as a recruiting signal for effectors such as the WD-repeat domain phosphoinositide-interacting (WIPI) proteins (12, 13). Both the ULK1 complex and PI3KC3-C1 are regulated by multiple ubiquitin signals.

Here, we show that KO of the ubiquitin-specific protease 11 (USP11) hyperactivates autophagy in human cell lines and the model organism *Caenorhabditis elegans*. Increased autophagic substrate degradation due to USP11 depletion in *C. elegans* protects against proteotoxicity and delays amyloid-β (Aβ) aggregation-induced paralysis of the worms. Mechanistically, our data show that USP11 restricts autophagy by limiting the interaction between PI3KC3-C1 and nuclear receptor-binding factor 2 (NRBF2), which controls lipid kinase activity and subsequent recruitment of effector proteins to the autophagosomal membrane. Furthermore, we demonstrate that USP11 regulates mTOR protein stability and thereby influences phosphorylation of mTORC1 substrates.

## Results

### USP11 regulates autophagic flux

**Figure 1:**
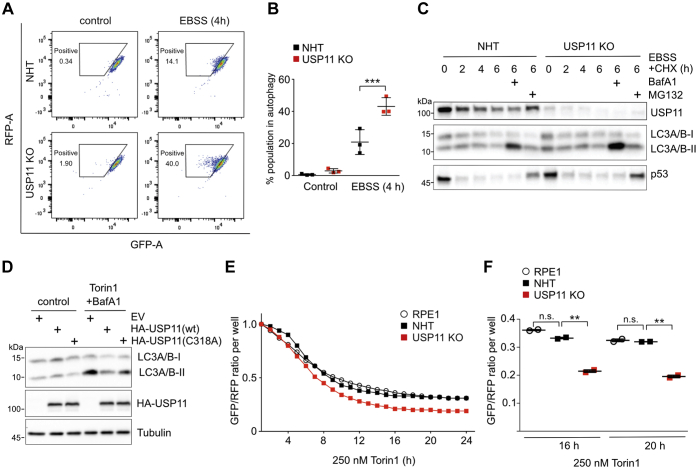
**USP11 regulates autophagic flux.***A*, scatter plot showing NHT control and USP11 KO RPE1 cell populations (single clones) expressing the GFP-LC3-RFP-LC3ΔG autophagy probe. USP11 KO decreased GFP/RFP ratios upon amino acid starvation (4 h EBSS treatment), indicative of higher autophagic flux. *B*, quantification of autophagy-positive cells based on gates shown in *panel A* revealed a statistically significant increase in autophagy in USP11 KO cells as compared with NHT control cells (∗∗∗*p*-value < 0.001, two-way ANOVA, and Bonferroni posttest, N = 3). *C*, immunoblot analysis showing a time course of EBSS-induced autophagy and CHX-mediated translational inhibition. LC3-II levels were increased in USP11 KO cells as compared with NHT control cells after 6 h EBSS/CHX and bafilomycin A1 (BafA1) treatment. *D*, immunoblot analysis showing Torin 1-induced autophagy and bafilomycin A1-induced autophagosome accumulation in HA-USP11 wt or HA-USP11(C318A) expressing 293 cells. Overexpression of wt USP11 resulted in decreased LC3A/B lipidation as compared with the EV control, whereas inactive USP11 only had a mild effect on LC3A/B-II level. *E*, real-time quantitative live-cell imaging of NHT control and USP11 KO RPE1 cells, as well as RPE1 cells without any gRNAs, all expressing GFP-LC3-RFP-LC3ΔG autophagy probe (single clones). Average GFP/RFP ratios upon 250 nM Torin 1 treatment plotted over time revealed long-term increase in autophagic flux upon loss of USP11. *F*, quantification of time points 16 h and 20 h based on GFP/RFP ratios revealed statistical significance between NHT control and USP11 KO cells (∗∗*p*-value < 0.01, two-way ANOVA, and Bonferroni posttest, N = 2). No significant difference between RPE1 NHT control cells and gRNA-free RPE1 cells expressing the autophagy probe, confirming that the nonhuman targeting gRNA has no effect on autophagy (two-way ANOVA, and Bonferroni posttest). CHX, cycloheximide; EBSS, Earle's balanced salt solution; EV, empty vector; LC3-II, phosphatidylethanolamine-conjugated LC3; NHT, nonhuman targeting; n.s., not significant; USP11, ubiquitin-specific protease 11.

To study the role of USP11 in autophagy regulation, we used CRISPR-Cas9 technology to establish USP11 KO and nonhuman targeting (NHT) control human hTERT RPE1 cell lines that stably expressed the autophagy flux probe GFP-LC3-RFP-LC3ΔG (14). This probe is cleaved by endogenous ATG4 proteases into equimolar amounts of GFP-LC3 and RFP-LC3ΔG. GFP-LC3 is degraded by autophagy, whereas conjugation-deficient RFP-LC3ΔG remains in the cytosol and serves as an internal control. We monitored GFP and RFP fluorescent signals in control and USP11 KO cells by flow cytometry (Fig. 1*A*) and calculated the GFP-RFP signal ratio. Loss of USP11 significantly increased autophagic flux upon amino acid starvation (Fig. 1*B*). Transient knockdown of USP11 using two different siRNAs in RPE1 cells stably expressing the autophagy flux probe caused the same phenotype (Fig. S1). Complementarily, we determined the amount of phosphatidylethanolamine-conjugated LC3, which correlates with the number of autophagosomes (15). Autophagy was induced in control and USP11 KO cells by amino acid starvation, and the LC3A/B protein level was followed up over time by Western blot analysis. To obtain information about the transit of LC3A/B-II through the autophagic pathway, we included bafilomycin A1 to inhibit lysosomal degradation and the translation inhibitor cycloheximide. Our data suggest that autophagic flux is increased in USP11-deficient cells as compared with control cells (Fig. 1*C*). In contrast, the LC3A/B-II level was decreased in cells overexpressing HA-tagged USP11 as compared with control cells, whereas inactive HA-USP11(C318A) only had a mild effect (Fig. 1*D*). To improve the temporal resolution of the USP11-dependent autophagy regulation, time course flux analyses were performed using live-cell imaging. We included parental RPE1 cells as well as NHT and USP11 KO RPE1 cells, all of which expressed the autophagy flux probe. GFP and RFP fluorescent signals were determined for 24 h in the presence of the mTOR kinase inhibitor Torin 1, a potent inducer of autophagy (Fig. 1, *E* and *F*). Under these experimental conditions, loss of USP11 accelerated the turnover of GFP-LC3 as shown by decreased GFP-RPF signal ratios, which confirms an increased autophagic flux. Of note, no statistically significant differences were observed between parental and NHT RPE1 cells, highlighting that our procedure for establishing CRISPR-Cas9–mediated KO cells did not induce autophagy *per se*. Taken together, our data obtained from complementary assays provided strong evidence that USP11 restricts autophagy.

### USP11 regulates autophagy in *C. elegans*

**Figure 2:**
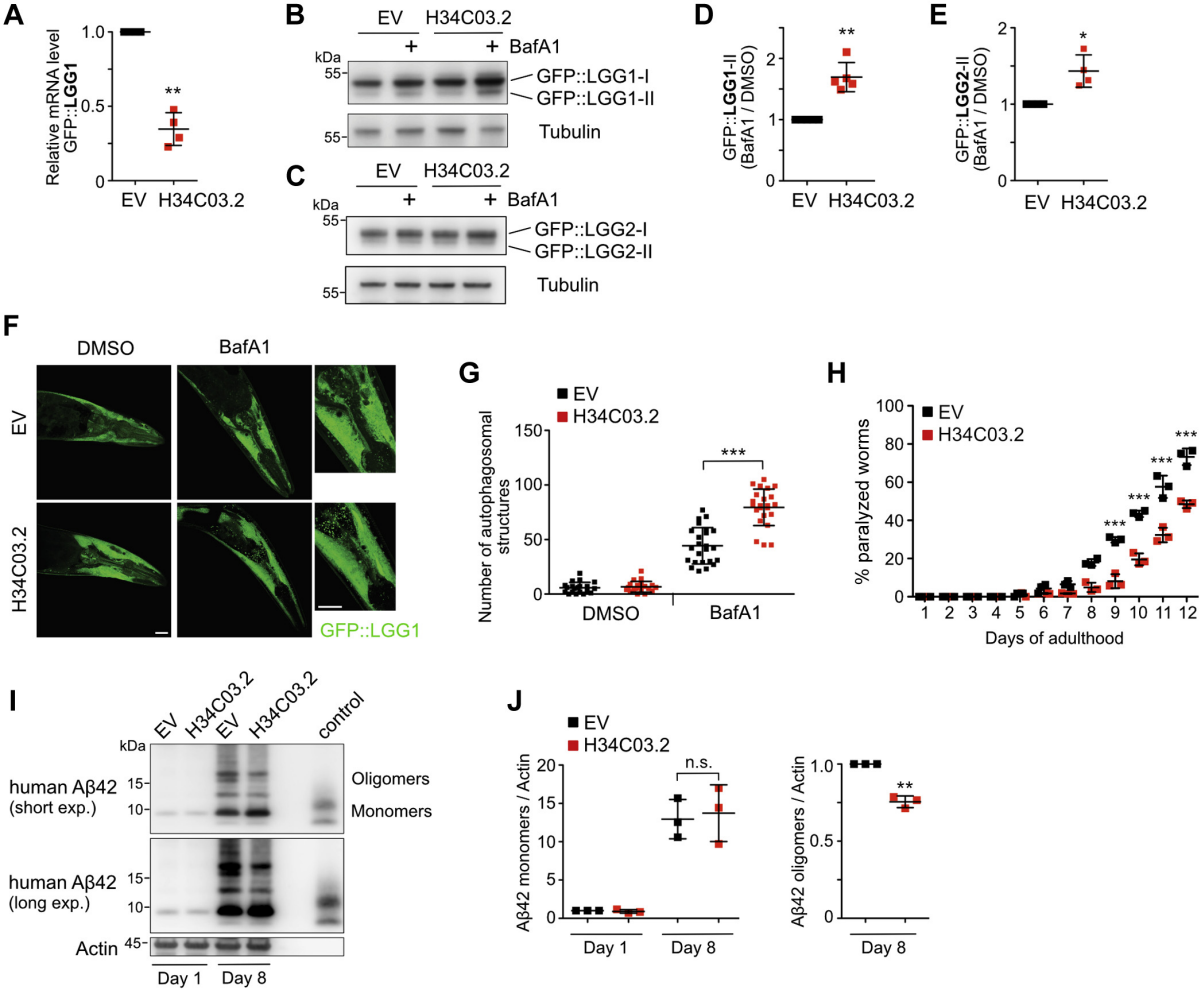
**Knockdown of USP11 orthologue H34C03.2 in *Caenorhabditis elegans* promotes autophagy.***A*, RNAi-mediated depletion of H34C03.2 was assessed by qPCR. Quantification demonstrated a statistically significant decrease in H34C03.2 mRNA levels as compared with EV control (∗∗*p*-value < 0.005, one-sample *t* test, N = 4). *B* and *C*, immunoblot analysis showing increased GFP::LGG1-II levels (*B*) or GFP::LGG2-II levels (*C*) accumulating upon BafA1 treatment in H34C03.2 knockdown worms as compared with EV control. *D*, quantification of immunoblots shown in panel B (∗∗*p*-value < 0.005, one-sample *t* test, N = 5). *E*, quantification of immunoblots shown in *panel C* (∗*p*-value < 0.05, one-sample *t* test, N = 4). *F*, confocal laser scanning microscopy displayed an increased number of GFP-LGG1 puncta after 2-h BafA1 treatment in H34C03.2 knockdown worms, indicative of a higher autophagosome number (the scale bar represents 50 μm). *G*, quantification of autophagosomal structures shown in *panel F*. GFP-positive structures in the head region of 20 to 23 individual worms from four independent experiments were quantified (∗∗∗*p*-value < 0.0001, two-sample *t* test). *H*, knockdown of H34C03.2 led to a delayed paralysis phenotype in *C. elegans* strain CL2006 expressing human amyloid-β protein 1 to 42 (hAβ42) in muscle cells. Quantification of days 8 to 12 of adulthood revealed the statistical significance of the observed phenotype (∗∗*p*-value < 0.01 for day 8, ∗∗∗*p*-value < 0.001 for days 9–12, two-way ANOVA, and Bonferroni posttest). *I*, immunoblot analysis showing hAβ42 expression in *C. elegans* strain CL2006 upon H34C03.2 knockdown. *J*, quantification of Aβ42 monomers (no significant difference between H34C03.2 knockdown worms and EV control) and oligomers at day 8 of adulthood (∗∗*p*-value < 0.01, one-sample *t* test, N = 3) BafA1, bafilomycin A1; EV, empty vector; n.s., not significant; USP11, ubiquitin-specific protease 11.

To investigate if USP11-dependent autophagy regulation is evolutionarily conserved, we included the nematode *C. elegans* in our studies. We depleted the USP11 homolog H34C03.2 in worms expressing the ubiquitin-like modifier GFP::LGG1, the homolog of human GABARAP or GFP::LGG2, the homolog of human LC3. To determine siRNA-mediated knockdown efficiency, relative mRNA levels of H34C03.2 were examined by qPCR analysis (Fig. 2*A*). Subsequently, we monitored autophagic activity by quantifying the flux of GFP::LGG1 or GFP::LGG2 in the presence and absence of bafilomycin A1 (Fig. 2, *B*–*E*). When lysosomal degradation was blocked, knockdown of H34C03.2 resulted in elevated levels of GFP::LGG1-II (Fig. 2, *B* and *D*) and moderately increased levels of GFP::LGG2-II (Fig. 2, *C* and *E*) as compared with the empty vector control, suggesting that USP11 (H34C03.2) also negatively regulates the autophagic flux in *C. elegans*. This conclusion was supported by monitoring GFP-LGG1 by confocal fluorescence microscopy. Image analysis revealed significantly more GFP-positive puncta in H34C03.2-depleted worms upon bafilomycin A1 treatment than in control animals, indicating a higher number of autophagosomes (Fig. 2, *F* and *G*). Autophagy is a housekeeping process responsible for the degradation of misfolded or aggregated proteins and damaged organelles, and it has been implicated in various proteotoxicity-associated disorders. We tested if increased autophagic activity due to USP11 depletion protects against proteotoxicity caused by Aβ. We used the transgenic *C. elegans* strain CL2006 expressing human amyloid-β protein 1 to 42 (hAβ42) under the control of a muscle-specific promoter (16). Aβ-induced paralysis in CL2006 worms is the consequence of disturbed protein homeostasis. We quantified paralyzed worms from day 1 of adulthood onward and observed that knockdown of H34C03.2 significantly delayed the paralysis phenotype observed in unperturbed CL2006 animals (Fig. 2*H*), pointing to a more productive autophagic process alleviating hAβ42 aggregation–induced paralysis. Correspondingly, less hAβ42 oligomers were detected in H34C03.2-depleted worm, while expression of hAβ42 monomers was comparable in control and knockdown animals (Fig. 2, *I* and *J*). In summary, our data showed that USP11 controls autophagic substrate degradation both in *C. elegans* and human cells. We established USP11 as a novel, evolutionarily-conserved negative regulator of the autophagy pathway.

### Multiple autophagy-related proteins interact with USP11

**Figure 3:**
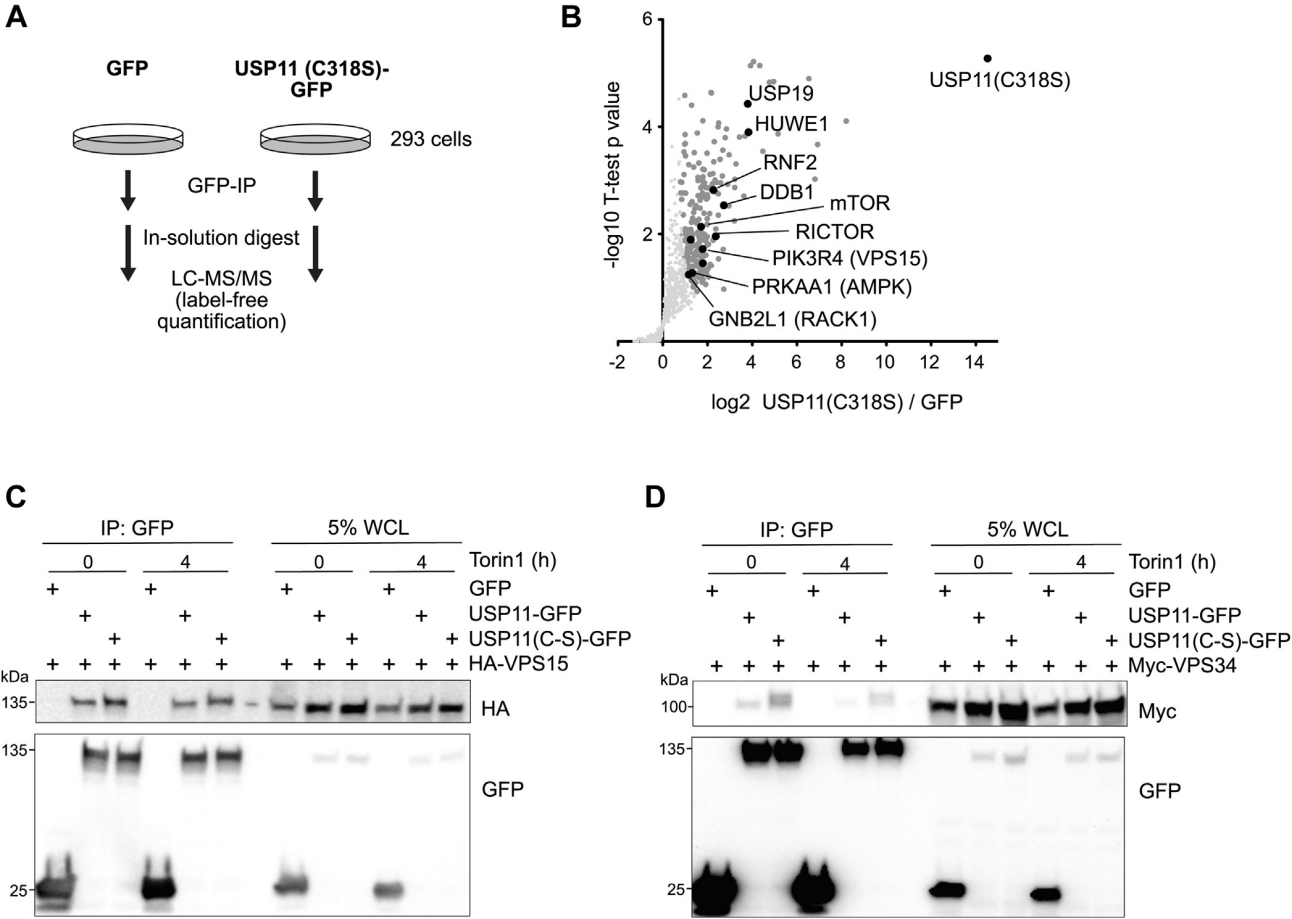
**USP11 interacts with multiple autophagy-related proteins.***A*, cartoon depicting design of affinity-based MS experiment for the identification of USP11 interactors. *B*, volcano plot of USP11(C318S)-GFP interactome over GFP upon 4-h Torin 1 treatment revealed multiple autophagy-related proteins. The strongest binding partners identified in this experiment (not labeled in the plot) comprised TCEAL1, ZNF598, USP7, MYCBP2, and BIRC6 (for more information, see Table S1). *C*, verification of the interactome analysis shown in panel B by Western blot analysis of coimmunoprecipitation of USP11-GFP or USP11(C318S)-GFP with HA-VPS15. VPS15 specifically coimmunoprecipitated with both variants of USP11, independent of autophagy induction by Torin 1. *D*, analogue to panel C, USP11-GFP and USP11(C318S)-GFP coimmunoprecipitated Myc-VPS34, the lipid kinase of the PI3KC3 complex. A stronger interaction between catalytic inactive USP11 and VPS34 was observed. PI3KC3, class III phosphatidylinositol 3-kinase; USP11, ubiquitin-specific protease 11.

To mechanistically understand how USP11 controls autophagy, we aimed to identify relevant substrates of this DUB. To this end, we performed label-free MS-based interactome studies to determine binding partners and potentially trapped substrates of catalytically inactive USP11(C318S) (Fig. 3*A*). Mutant USP11(C318S)-GFP was expressed in 293 cells, which were treated with Torin 1 for 4 h before cell lysis and subsequent sample preparation. We observed coprecipitation of multiple proteins related to various stages of the autophagy pathway (Fig. 3*B*, Table S1). Among them were several proteins described to regulate PI3KC3-C1, as well as VPS15 (PIK3R4), a component of PI3KC3-C1 itself. To confirm these interactions, GFP-tagged WT or inactive USP11 were expressed together with VPS15 or the lipid kinase VPS34 (PIK3C3) in 293 cells, and coimmunoprecipitation experiments were carried out both under basal conditions and upon autophagy induction. Whereas USP11 coprecipitated VPS15 independent of its catalytic activity (Fig. 3*C*), VPS34 seemed to bind inactive USP11(C318S) more efficiently (Fig. 3*D*). The appearance of more than one band hints at a potentially modified VPS34 trapped by inactive USP11(C318S). Proautophagic PI3KC3-C1 further comprises the regulatory subunit Beclin 1 and the early autophagy-specific targeting subunit ATG14. In addition, both proteins coprecipitated with USP11 (Fig. S2).

### USP11 impacts proautophagic PI3KC3-C1 activity

**Figure 4:**
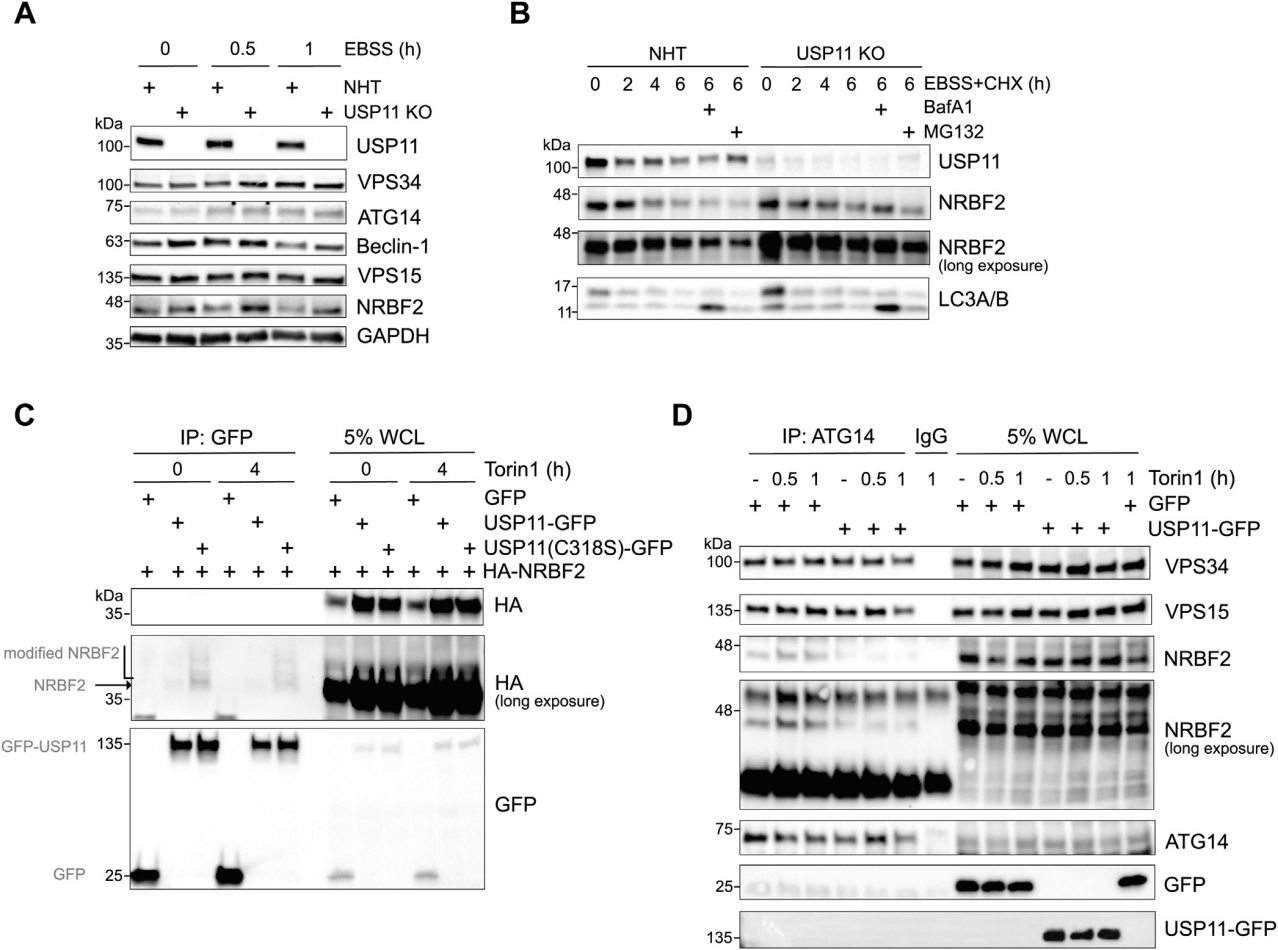
**USP11 regulates proautophagic PI3KC3 complex I via NRBF2.***A*, immunoblot determining protein stability of PI3KC3 complex I components upon autophagy induction demonstrated increased NRBF2 protein level in USP11 KO RPE1 cells. *B*, CHX chase confirming elevated NRBF2 stability in USP11 KO cells as compared with NHT control cells upon longer autophagy induction. As expected, LC3-II levels were increased in BafA1-treated USP11 KO cells compared with NHT control. *C*, coimmunoprecipitation of WT USP11-GFP or catalytic inactive USP11(C318S)-GFP and HA-NRBF2 in 293 cells suggested USP11 activity-dependent modification of NRBF2. *D*, immunoprecipitation of endogenous ATG14 from 293 cells upon overexpression of USP11-GFP revealed decreased binding of NRBF2 to ATG14. BafA1, bafilomycin A1; CHX, cycloheximide; LC3-II, phosphatidylethanolamine-conjugated LC3; NRBF2, nuclear receptor-binding factor 2; NHT, nonhuman targeting; PI3KC3, class III phosphatidylinositol 3-kinase; USP11, ubiquitin-specific protease 11.

Based on the determined interaction partners of USP11(C318S), we hypothesized that USP11 may impact autophagy via PI3KC3-C1. Expression levels of VPS15, VPS34, Beclin 1, and ATG14, both under fed and amino acid–starved growth conditions, were comparable in control and USP11 KO cells (Fig. 4*A*), suggesting that USP11 does not regulate proteasomal degradation of these proteins. However, we observed that NRBF2, the fifth subunit of PI3KC3-C1 (17, 18, 19), accumulated in USP11 KO cells when autophagy was induced (Fig. 4*A*). This result also argued against a proteasomal degradation signal on NRBF2 that would be reversed by USP11. Rather, we hypothesized that NRBF2 may be targeted by an E3 ligase that could be a direct substrate of USP11, given that various E3 ligases were identified in our USP11 interactome study (Fig. 3*B*, Table S1). Loss of USP11 would lead to degradation of this candidate E3 ligase or its impaired activity, resulting in reduced NRBF2 ubiquitination and stabilized NRBF2 levels. Alternatively, USP11 may directly regulate a nonproteolytic signal on NRBF2 that could promote interaction between NRBF2 and the other subunits of PI3KC3-C1. The hypothesized modification could protect NRBF2 from extraction from the complex and subsequent degradation. Thereby, USP11 could potentially regulate complex formation and its activity.

To test these hypotheses, we first induced autophagy by amino acid starvation and followed NRBF2 level over time. In USP11 KO cells, NRBF2 turnover appeared slower than in NHT control cells (Fig. 4*B*). Because the growth medium was supplemented with cycloheximide to inhibit the synthesis of new proteins, regulation of NRBF2 transcription or translation by USP11 was unlikely. Interestingly, GFP-tagged, catalytic inactive USP11(C318S) coprecipitated more NRBF2 than WT USP11 (Fig. 4*C*). Furthermore, the appearance of multiple NRBF2 bands, which are indicative of modified protein species, supported our hypothesis that USP11 directly counteracts NRBF2 ubiquitination. To address the question if USP11-regulated ubiquitination of NRBF2 influences its interaction with PI3KC3-C1, endogenous ATG14 was immunoprecipitated from 293 cells expressing GFP-tagged USP11 or GFP alone, and binding of other complex components was determined by Western blot (Fig. 4*D*). In the presence of USP11, less NRBF2 coprecipitated with ATG14 as compared with control cells, suggesting that USP11 impacts PI3KC3-C1 activity via NRBF2, whose interaction with the complex was shown to enhance the lipid kinase activity of the catalytic subunit VPS34 (20, 21).

**Figure 5:**
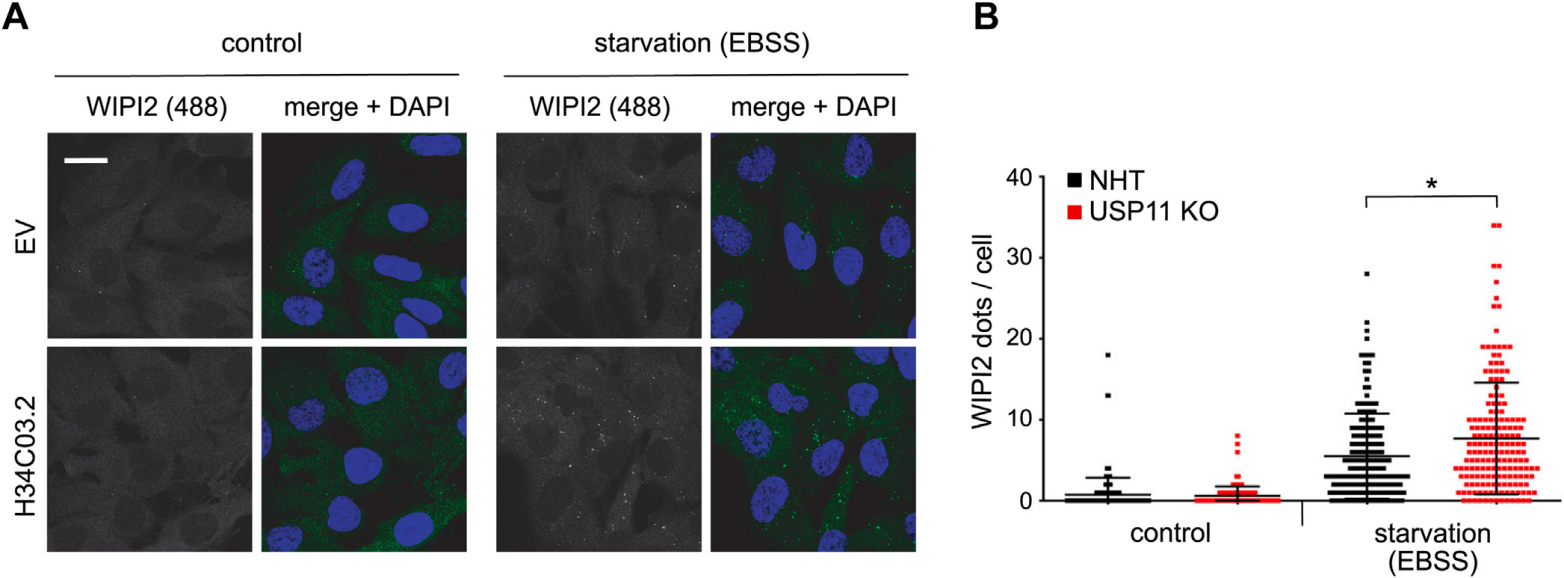
**USP11 KO increases WIPI2 dot formation.***A*, IF of endogenous WIPI2 showed increased dot formation in USP11 KO cells as compared with NHT control cells after 1-h EBSS treatment indicative of increased activity of PI3KC3 complex I. The scale bar represents 20 μm. *B*, quantification of IF images shown in *panel A* (∗*p*-value < 0.05, two-sample *t* test, approximately 450 cells per sample, per condition (N = 3)). EBSS, Earle's balanced salt solution; IF, immunofluorescence; NHT, nonhuman targeting; PI3KC3, class III phosphatidylinositol 3-kinase; USP11, ubiquitin-specific protease 11; WIPI2, WD-repeat domain phosphoinositide-interacting 2.

PI3KC3-C1 is essential for the initiation of autophagosomes. VPS34 generates the lipid PI(3)P, which is recognized by downstream effector proteins such as WIPI2. Upon autophagy induction, WIPI2 forms distinct dots that can be quantified by immunofluorescence using an antibody against endogenous WIPI2 protein. This approach is an indirect method to determine PI3KC3-C1 lipid kinase activity (22). In accordance with our previous data, USP11 deficiency resulted in more WIPI2-positive dots per cell upon autophagy induction (Fig. 5), indicating more active PI3KC3-C1. Taken together, we showed that USP11 interacts with multiple components of the autophagy machinery. Our data suggested that USP11 regulates PI3KC3-C1 activity by limiting NRBF2 binding to the complex. Increased WIPI2 recruitment to the growing autophagosomal membrane in USP11 KO cells confirmed an increased lipid kinase activity of PI3KC3-C1.

### USP11 controls mTOR stability

**Figure 6:**
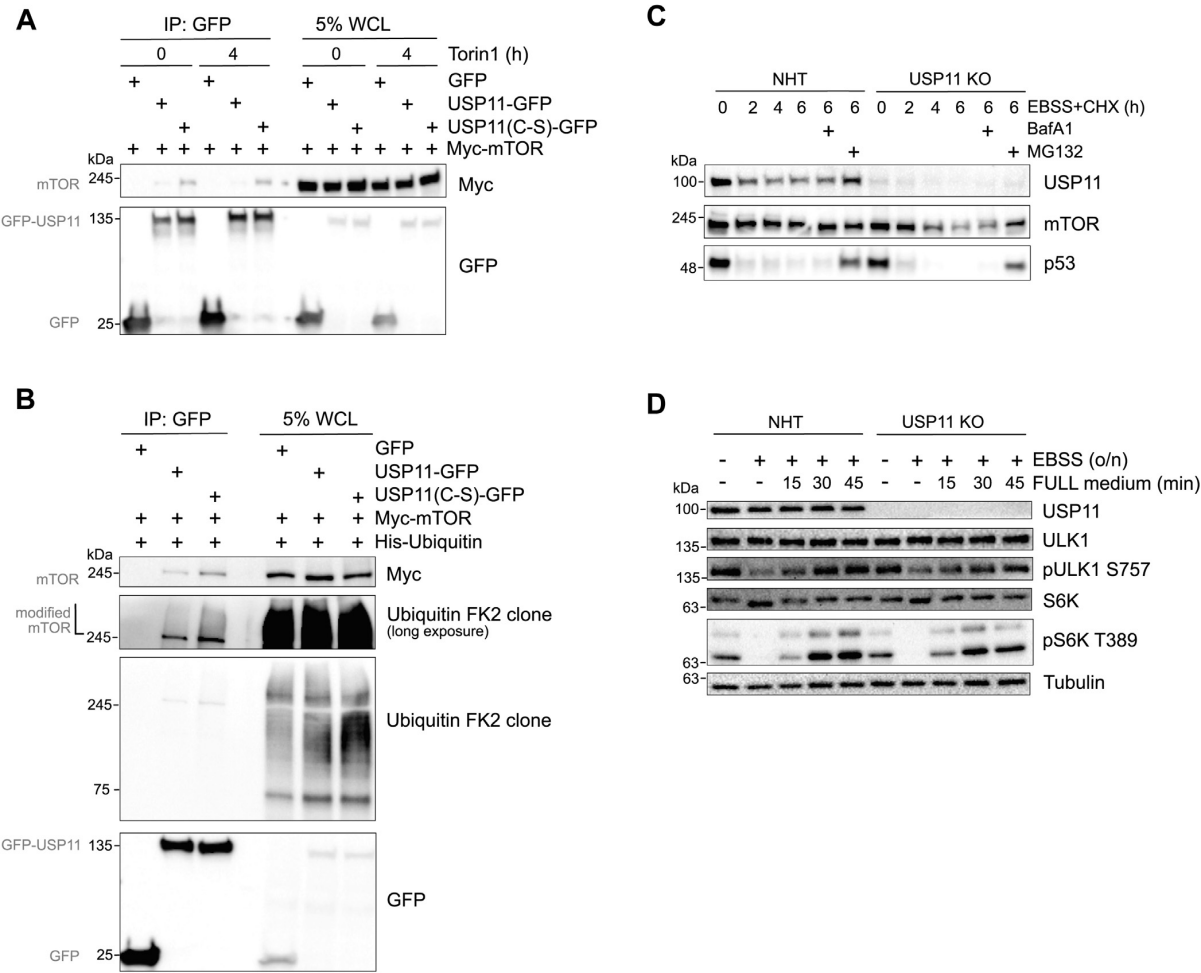
**USP11 controls mTOR stability.***A*, coimmunoprecipitation of USP11-GFP and myc-mTOR in control or Torin 1-treated 293 cells confirmed interaction of the proteins as suggested by MS data (Fig. 3). Catalytic inactive USP11(C318S) coprecipitated more mTOR than the WT DUB, suggesting that mTOR is a potential substrate of USP11. *B*, coimmunoprecipitation of USP11-GFP and myc-mTOR in control or Torin 1-treated 293 cells expressing His-ubiquitin. Smeared appearance of the mTOR band suggested increased levels of ubiquitinated mTOR when coprecipitated with catalytic inactive USP11(C318S). *C*, CHX chase revealed a higher turnover of mTOR protein in USP11 KO RPE1 cells than NHT control cells. Protein levels were rescued with MG132 treatment, indicating that USP11 prevented proteasomal degradation of mTOR. *D*, mTOR-dependent substrate phosphorylation after reactivation of the kinase was assessed by Western blot analysis. CHX, cycloheximide; DUB, deubiquitinase; mTOR, mechanistic target of rapamycin; NHT, nonhuman targeting; USP11, ubiquitin-specific protease 11.

Interestingly, our interactome study also identified mTOR as a binding partner of USP11 (Fig. 3*B*). The serine/threonine-protein kinase is part of the mTORC1 complex that negatively regulates autophagy. To verify the interaction between both proteins, GFP-tagged WT or catalytic inactive USP11 were expressed together with mTOR in 293 cells, and coimmunoprecipitation experiments were carried out both under basal conditions and upon Torin 1 treatment (Fig. 6*A*). mTOR coprecipitated with USP11 independent of autophagy induction by Torin 1. However, more mTOR seemed to interact with inactive USP11(C318S) than with the WT DUB, pointing to mTOR as a potential substrate of USP11. To further investigate this observation, the same coimmunoprecipitation was performed in the presence of His-tagged ubiquitin to amplify a potential mTOR modification (Fig. 6*B*). Enhanced binding between mTOR and USP11(C318S) could be confirmed. In addition, a ubiquitin-specific antibody detected smeared mTOR bands, which were more prominent in the presence of USP11(C318S) (Fig. 6*B*), indicating a potential ubiquitination of mTOR. This effect was even stronger when the proteasome inhibitor MG132 was included (Fig. S3*B*). To determine if USP11-controlled mTOR ubiquitination results in proteasomal degradation of the kinase, the mTOR level was monitored upon autophagy induction in the presence of cycloheximide. Our data showed that mTOR protein was turned over faster in USP11 KO cells (Fig. 6*C*), an effect that was rescued by MG132, supporting a USP11-dependent regulation of mTOR stability. Subsequently, we addressed whether altered mTOR stability affects activity of mTORC1 in USP11 KO cells. To this end, phosphorylation of mTORC1 substrates was analyzed in control and USP11 KO cells after reactivation of the kinase by transferring cells from an amino acid–depleted culture medium to a full medium (Fig. 6*D*). Both 30 and 60 min after reactivation of mTORC1, phosphorylation of ribosomal protein S6 kinase (S6K) at T389 and the autophagy-relevant kinase ULK1 at S757 was decreased in the absence of USP11. These results showed that USP11 also impacts autophagy induction by modulating mTOR stability and consequential mTORC1 activity.

## Discussion

The ubiquitin proteasome system and autophagy are two major quality control mechanisms for proteins and organelles, together forming an interconnected network to achieve homeostasis (23). Autophagic substrate degradation itself is tightly controlled by ubiquitination, and multiple E3 ligases and DUBs have been identified in this context (4). Here, we show that USP11 impacts autophagy in human cells and *C. elegans*, establishing it as an evolutionarily conserved regulator. Most strikingly, in a *C. elegans* model of disturbed proteostasis and proteotoxicity, driving autophagy by depletion of USP11 protected the worms against Aβ toxicity–induced paralysis.

Interestingly, USP11 has already previously been recognized as a member of the autophagy interaction network (24). This study showed binding between various bait proteins of the autophagy system, for example, PIK3C3 (VPS34), ATG12, ATG4B, or LC3B and endogenous USP11, which supports our model of USP11 as a multifunctional player in the autophagy pathway. It also indicates that the numerous autophagy-relevant interactions that we observed in our interactome study are not an artifact caused by overexpression of GFP-tagged USP11.

We addressed the role of USP11 in the context of autophagy-initiating PI3KC3-C1. PI3KC3-C1 is extensively regulated by posttranslational modifications, especially by phosphorylation and ubiquitination (4), but also SUMOylation and acetylation of complex components have been described (25, 26). In addition to different E3 ubiquitin ligases that modify PI3KC3-C1, several DUBs have already been shown to control and terminate these ubiquitin signals (8). Our work revealed USP11 as a new DUB associated with PI3KC3-C1. USP11 does not control protein stability of the complex components Beclin-1, VPS15, VPS34, or ATG14. Rather, our data support a regulatory ubiquitin signal on NRBF2 that is counteracted by USP11, and that has a positive effect on the interaction between NRBF2 and heterotetrameric PI3KC3-C1 upon autophagy induction, and ultimately on lipid kinase activity.

The overall architecture of PI3KC3-C1 has the shape of the letter V (27). Structural analyses showed that VPS15 restricts the activation loop of VPS34 and that major structural changes are necessary for full VPS34 activity (20, 21). Binding of NRBF2 at the base of the V triggers rearrangements in the right arm of the complex, leading the lipid kinase domain to adopt a conformation with an unrestricted activation loop and an active site poised to phosphorylate membrane-embedded phosphatidylinositol (21). Thus, the restrictive effect of USP11 on autophagy could partly be mediated by reducing the interaction between NRBF2 and PI3KC3-C1.

In the framework of the presented study, a potential nonproteolytic, USP11-regulated ubiquitination of VPS34 (as suggested by Fig. 3*D*; additional VPS34 bands in lanes 3 and 6) has not been addressed. However, it is thinkable that such a modification on VPS34 promotes lipid kinase activity by interfering with the inactive conformation. This possibility needs to be further investigated. In addition, it will be interesting in future to address the interplay between the different ubiquitin signals attached to PI3KC3-C1, and between the enzymes that mediate and regulate them.

We also identified an interaction between USP11 and mTOR; our data suggested that USP11 regulates mTOR stability upon autophagy induction by amino acid starvation. The serine/threonine–protein kinase mTOR is one of the key metabolic regulators involved in almost any signaling pathway from lysosomal biogenesis via transcription factor EB in mTORC1 to cytoskeleton rearrangement via PKCα in mTORC2 (9). For autophagy regulation, only mTORC1 is relevant, and it is defined by its accessory proteins mLST8 and Raptor. In mTORC2, Raptor is replaced by RICTOR. We observed decreased mTORC1-dependent phosphorylation of substrate proteins in USP11 KO cells upon reactivation of the kinase complex after amino acid starvation. The slower reactivation of mTORC1 could be a consequence of the lower mTOR abundance but could also be due to an allosteric effect mediated by the interaction with USP11.

Interestingly, our interactome analysis also showed that RICTOR coprecipitated with USP11, implying that USP11 can associate with both mTORC1 and mTORC2. Signaling of both kinase complexes is highly interconnected, and published data indicate that USP11 is involved in both mTORC1- and mTORC2-dependent signaling pathways via PTEN (28). However, a specific interaction between USP11 and mTOR has not been described before.

Active mTORC1 regulates protein translation by phosphorylating ribosomal protein S6K, subsequently activated further by PDK1 (29), leading to S6K-mediated phosphorylation of the members of the translation initiation complex, among which is eIF4B (30). Upon S6K-mediated phosphorylation, eIF4B dissociates from both S6K and mTOR, leading to translation initiation. Interestingly, S6K was shown to phosphorylate USP11 at S453, which promotes USP11 and eIF4B interaction, and the stabilization of eIF4B by USP11 deubiquitination, thereby driving translation initiation (31). This observation directly links USP11 and mTORC1-mediated translation regulation and places USP11 on the same signaling axis as active mTORC1, which indicates conditions under which autophagy is suppressed. Ma *et al*. [32] described mTORC1-mediated phosphorylation of NRBF2. The authors showed that NRBF2 phosphorylation is reduced upon amino acid starvation or mTORC1 inhibition. Their data implied that NRBF2 in its unphosphorylated form promotes VPS34 lipid kinase activity and autophagy flux, whereas its phosphorylated form blocks them (32). Hence, USP11 would also indirectly influence PI3KC3-C1 activity via its effect on mTORC1 activity and therefore the NRBF2 phosphorylation status.

In summary, USP11 is a versatile regulator of the autophagy pathway that keeps the degradation process in balance. Cells that lack USP11 show elevated autophagic flux, a phenotype that cannot be pinned down to a single substrate of USP11 that is no longer deubiquitinated. Rather, it seems that USP11 impinges on the autophagy pathway at multiple sites, and many contact points have not been investigated yet. Future work will reveal more details of the regulatory mechanisms via which USP11 restricts autophagy, which is of broader interest because our data raised the possibility that autophagy activation by inhibiting USP11 could be a therapeutic option to alleviate symptoms of proteotoxicity, which is a major hallmark of neurodegenerative diseases.

## Experimental procedures

### Cell culture

hTERT-RPE1 cells were obtained from the American Type Culture Collection (ATCC CRL-4000) and grown in Dulbecco's modified Eagle's medium (DMEM)/F-12, GlutaMAX medium (Gibco) supplemented with 10% (v/v) fetal bovine serum (Gibco), 200 μg/ml Hygromycin B, 50 U/ml penicillin, and 50 μg/ml streptomycin (GE Healthcare) at 37 °C and 5% CO_2_. U2OS, 293, and 293T cells were obtained from the Leibniz Institute DSMZ-German Collection of Microorganisms and Cell Cultures (DSMZ no. ACC 785, ACC 305, and ACC 635, respectively) and grown in DMEM, GlutaMAX medium (Gibco) supplemented with 10% (v/v) fetal bovine serum (Gibco), and 50 U/ml penicillin and 50 μg/ml streptomycin (GE Healthcare) at 37 °C and 5 % CO_2_. PCR-based *Mycoplasma* contamination tests were regularly performed using the VenorGeM Classic kit (Minerva Biolabs). Autophagy was induced by amino acid starvation using EBSS medium (Gibco).

### Antibodies

The following antibodies were used in this study: ATG14 (Cell Signaling Technology, 5504, 1:1000), Beclin-1 (D40C5) (Cell Signaling Technology, 3495, 1:1000), GAPDH (14C10) (Cell Signaling Technology, 2118, 1:1000), GFP (B-2) (Santa Cruz Biotechnology, sc-9996, 1:10,000), HA-tag (6E2) (Cell Signaling Technology, #2367, 1:1000), LC3A/B (D3U4C) XP (Cell Signaling Technology, #12741, 1:1000), mTOR (7C10) (Cell Signaling Technology, #2983, 1:1000), multiubiquitin chain (Clone FK2) (Cayman Chemical, #14220, 1:1000), myc-tag (9B11) (Cell Signaling Technology, #2276, 1:1000), PI3K class III (D4E2) (Cell Signaling Technology, #3358, 1:1000), p70 S6 kinase (Cell Signaling Technology, #9202, 1:1000), phospho-p70 S6 kinase (T389) (Cell Signaling Technology, #9205, 1:1000), tubulin (Cell Signaling Technology, #2125, 1:1000), ULK1 (D8H5) (Cell Signaling Technology, #8054, 1:1000), phospho-ULK1 (S757) (Cell Signaling Technology, #6888, 1:1000), USP11 (Sigma-Aldrich, HPA003103, 1:1000), Vinculin (Sigma-Aldrich, V4505, 1:10,000), and VPS15 (Bethyl, A302-571A-M, 1:1000).

Secondary antibodies used in this study were obtained from Cell Signaling Technology: anti-mouse IgG-HRP (7076, 1:2500) and anti-rabbit IgG-HRP (7074, 1:2500), or Abcam: anti-rabbit IgG light-chain HRP (99697, 1:10,000). All primary antibodies were diluted in 3% bovine serum albumin (BSA) (prepared in TBST, 0.05% Tween-20, and 0.05% sodium azide). Secondary antibodies were diluted in 2.5% BSA (prepared in TBST).

### Inhibitors

The following inhibitors were used in this study: bafilomycin A1 (200 nM), chloroquine (50 μM), cycloheximide (100 μg/ml), MG132 (10 μM), Torin 1 (250 nM), and KU-0063794 (10 μM).

### Plasmids, cloning, and site-directed mutagenesis

Oligonucleotide primers were designed with In-Fusion Cloning Primer Design Tool (Clontech) and purchased from Sigma-Aldrich. pIREpuro2-HA-USP11(wt), pIREpuro2-HA-USP11(C318A), and pIREpuro2-HA-NRBF2 were generated using the In-Fusion cloning system (In-Fusion HD Cloning Kit, Clontech) according to manufacturer's instructions. Further plasmids used in this study are as follows: pMRX-IP-GFP-LC3-RFP-LC3ΔG (gift from Noboru Mizushima, Addgene, plasmid #84572), pcDNA3-myc-PIK3C3 and pRK5-myc-mTOR (gifts from Ivan Dikic), and pBSSK-8× His-ubiquitin (gift from Stefan Müller).

#### Mutagenesis primer USP11(C318S)

5′-CTCACCAATCTGGGCAACACGTCCTTCATGAACT CGGCCCTGCAG-3′ was generated using QuikChange Primer Design Tool (Agilent) and used to generate catalytic inactive USP11 using Phusion High-Fidelity DNA Polymerase (NEB, M0530S) according to manufacturer's instructions.

### RNAi

siRNA oligonucleotides were purchased from Dharmacon/Horizon (USP11: J-006063-06, J-006063-08) and QIAGEN (nontargeting control: 1027281). Transfection was performed using Lipofectamine RNAiMAX (Invitrogen, 13778075) following a reverse-transfection protocol whereby 20 μM siRNA diluted in 200-μl prewarmed Opti-MEM (Invitrogen/Life Technologies) was pipetted into a 6-well plate onto which 2 × 10^5^ RPE1 cells were added and cultivated for 48 h.

### Guide RNA design and CRISPR/Cas9 plasmid generation

USP11 KO RPE1 cells were generated using the CRISPR/Cas9 technology. Guide RNA sequences targeting spCas9 to the genomic locus of USP11 (Ensembl ID: ENSG00000102226) were designed according to (33). Specific overhangs for subsequent ligation into pLentiCRISPRv2 (gift from Feng Zhang, Addgene, plasmid #52961) were added to each guide (underlined):

USP11_KO-1-F: CACCGggtctccatgatgatcaact

USP11_KO-1-R: AAACagttgatcatcatggagaccC

USP11_KO-2-F: CACCGgtgggcgagaacgtccactg

USP11_KO-2-R: AAACcagtggacgttctcgcccacC

USP11_KO-3-F: CACCGtgataggcagtggaacactg

USP11_KO-3-R: AAACcagtgttccactgcctatcaC

Complementary oligonucleotides were annealed for 5 min at 95 °C and subsequently cooled down for 15 min at room temperature (RT). Annealed primers were diluted to 0.5 μM in nuclease-free water and cloned into pLentiCRISPRv2 via BsmBI restriction enzyme (NEB) digest and subsequent ligation with T4 DNA ligase (NEB). Stellar competent cells (Clontech) were transformed with the ligation reaction, and correct clones were identified by Sanger sequencing (Microsynth Seqlab) using the U6 primer.

### Generation of high-titer lentivirus and viral transduction

The 293T cells (7.5 × 10^5^) were seeded into a 6-well plate and cultivated in DMEM without antibiotics 24 h before transfection. Cells were transfected with Lipofectamine 2000 (Invitrogen) by mixing the reagent with 200-μl Opti-MEM and 3.3-μg transfer vector containing the gRNAs (pLentiCRISPRv2), 2.7-μg PAX2 (a gift from Didier Trono, Addgene, plasmid #12260), and 1-μg pMD2.G (a gift from Didier Trono, Addgene, plasmid #12259). For establishing cell lines stably expressing the autophagy flux probe GFP-LC3-RFP-LC3ΔG (14), 8 × 10^5^ 293T cells were seeded. The cells were transfected with Lipofectamine 2000 (Invitrogen) by mixing the reagent with 200-μl Opti-MEM and plasmids in ratio 1:1:3 PCG Pol:SVG:GFP-LC3-RFP-LC3ΔG plasmid for a total of 3-μg DNA. The transfection mix was incubated for 30 min at RT and afterward added dropwise to 293T cells. The medium was replaced with fresh DMEM containing 10% (v/v) fetal bovine serum (Gibco) and 50 U/ml penicillin and 50 μg/ml streptomycin (GE Healthcare) 12 h after transfection. The supernatant containing lentiviral particles was collected after 24 h and 48 h. The supernatants were pooled and frozen at −80 °C.

For viral transduction, the supernatants were thawed at RT, sterile-filtered through 0.45-μm filters, and mixed with 10-μg polybrene (Sigma-Aldrich) to infect 1 × 10^6^ RPE1 cells. Stable transduced cells were selected with puromycin, and efficiency of USP11 KO cells was confirmed by immunoblotting using the antibody against USP11.

### Immunoblot

Proteins were separated on precast Mini-PROTEAN TGX gradient gels (4–15% or 4–20%, Bio-Rad) or self-made Tris-glycine gels and transferred (200 mA for 90 min, or 55 V for 75 min) onto 0.45-μm Immobilon-IP polyvinylidene fluoride membranes (Millipore) or 0.45-μm nitrocellulose membranes (Millipore) using the Mini Trans-Blot Cell System (Bio-Rad). Protein Marker VI (10–245 kDa) prestained (AppliChem) was used. Membranes were blocked with 2.5% BSA in TBST (50 mM Tris HCl (pH 7.6), 150 mM NaCl, 0.05% Tween-20). Blots were incubated overnight at 4 °C with primary antibodies. Blots were washed three times (each 5 min) with TBST and incubated for 1 h at RT with secondary antibodies (HRP-conjugated for chemiluminescence). Subsequently, blots were washed twice with TBST and once with TBS (50 mM Tris HCl (pH 7.6) and 150 mM NaCl). For chemiluminescence visualization, blots were incubated with the ECL Prime Western blotting detection reagent (GE Healthcare) and detected with ChemiDoc Imaging System (Bio-Rad).

### Flow cytometry

Cells were grown in 6-well plates, washed with PBS, and trypsinized in 100-μl 0.05% Trypsin-EDTA. The cells were collected in 1-ml PBS supplemented with 1% FBS and passed through the cell strainer cap of the FACS test tubes (Falcon). The cells were kept on ice until measurement on FACSAria FUSION cell sorter (Becton Dickinson). Data were analyzed, and figures were created using FlowJo (FlowJo, LLC).

### Live-cell imaging

RPE1 cells (7 × 10^4^) stably expressing the autophagy flux probe GFP-LC3-RFP (gift from Paolo Grumati) or RPE1 NHT and USP11 KO cells expressing GFP-LC3-RFP-LC3ΔG were seeded in a 96-well plate (Thermo) and grown for 18 h. Subsequently, the cells were treated with media containing 0.1% dimethyl sulfoxide or 250 nM Torin 1 and monitored in the IncuCyte live-cell analysis system (Sartorius) for 20 h. The cells were scanned at indicated time points for phase contrast and green/red fluorescence to obtain information about cell confluence and autophagic flux, respectively. Exported metadata were analyzed using Prism (GraphPad Software, Inc).

### Immunofluorescence

For immunofluorescence microscopy, cells were grown on uncoated glass coverslips in 6-well plates. After treatment, as indicated, cells were fixed with cold 4% paraformaldehyde solution in PBS for 15 min, washed twice with PBS, and permeabilized with 1% Triton X-100 in PBS for 15 min followed by a final wash with PBS. Samples were blocked with 2.5% BSA in PBS with 0.05% Tween-20 (BSA–PBS-T) for 30 min and incubated with the primary antibody solution (anti-WIPI2, 1:250 dilution) for 1 h at RT. Afterward, samples were washed 3 × 5 min with PBS to remove the residual antibody. Subsequently, the coverslips were incubated in the secondary antibody solution (anti-mouse-Alexa 488, 1:200 dilution) for 1 h at RT, shielded from light. After additional washing with PBS (3 × 5 min), cells were mounted using ProLong Diamond Antifade Reagent with DAPI (Molecular Probes).

All images were acquired on a confocal Leica SP8 LSM with a 63× oil-immersion objective in 1024 by 1024 scanning format using the standard Leica LAS-X software. Color channels were saved separately per image in TIF format for postcollection processing. Quantification of the number of dots was performed with the open-source cell image analysis software CellProfiler (version 3.1.9) (34).

Brightness and contrast were increased for all channels and conditions uniformly across the entire image using ImageJ (35), where necessary, for better visibility in the final figure.

### Immunoprecipitation

Immunoprecipitation (IP) was performed by seeding 293 cells in a 10-cm cell culture dish, followed by transfection 24 h later and cell collection 24 h after transfection. Transfection was performed using PEI (Polysciences; 23966-2) by mixing the reagent with 200-μl Opti-MEM and plasmids in the ratio 3:1. The transfection mix was incubated for 10 min at RT and afterward dropwise added to 293T cells. Cells were lysed on ice in the IP buffer (20 mM Tris HCl pH 7.5, 150 mM NaCl, 1% NP-40, 0.5% Triton-X, 1.5 μM Aprotinin, 100 μM Leupeptin, 1 mM PMSF, and 10 mM NaF) for 20 min and spun down, and the supernatant was transferred in a new tube. 10% input was taken, mixed with 4 × LDS buffer, and boiled at 95 °C for 3 min. The remaining sample was incubated with 10-μl prewashed GFP-Trap or Myc-Trap beads (Chromotek) and incubated with rotation at 4 °C for 1 h. The immunoprecipitate was washed 3 times, and the beads were mixed with 2× LDS buffer and boiled at 95 °C for 10 min.

Endogenous ATG14 IP was performed by seeding RPE1 cells in 10-cm cell culture dish and harvested 48 h later after indicated treatments. The cells were lysed in ATG14 IP buffer (20 mM Tris HCl, pH 7.5, 150 mM NaCl, 0.05% NP-40, and cOmplete, EDTA-free protease inhibitor cocktail (Roche)) for 30 min with rotation at 4 °C and spun down, and the supernatant was transferred into a new tube. 10% input was taken, mixed with 4 × LDS buffer, and boiled at 95 °C for 3 min. The remaining sample was incubated with 5 μl of ATG14 antibody or 5-μl normal rabbit IgG antibody and incubated for 1 h with rotation at 4 °C. 20 μl of prewashed SureBeads Protein A Beads (Bio-Rad) was added to the immunoprecipitates and incubated for 1 h at RT. The immunoprecipitates were washed with ATG14 IP lysis buffer and boiled in 2 × LDS buffer at 95 °C for 10 min.

### MS

USP11 C318S interactome was performed in three biological replicates following the protocol for the GFP-Trap IP. After the final IP wash, 20-μl elution buffer (50 mM ammonium bicarbonate, 2% Na-deoxycholate, 5 mM tris(2-carboxyethyl)phosphine, and 20 mM chloroacetamide) was added directly to the beads, and samples were boiled for 10 min at 95 °C. The cooled supernatants were transferred to new microtubes and incubated with 0.5 μg LysC for 3 h at 37 °C and 850 rpm. Trypsin (0.5 μg) in ammonium bicarbonate was added and further incubated at 37 °C and 850 rpm. Digested samples were mixed with isopropanol and TFA to stop the digestion and directly loaded on in-house assembled SDB-RPS StageTip. After two wash steps (0.1% TFA in isopropanol and then 0.1% TFA in water), peptides were eluted (5% ammonium hydroxide in 80% acetonitrile (ACN)), desiccated in a vacuum centrifuge, and rehydrated in 1% formic acid and 2% ACN.

The tryptic peptides were analyzed with Q Exactive HF coupled to an easynLC 1200 (ThermoFisher). In brief, the peptides were loaded onto a self-assembled 20-cm C18 column (1.7-μm particles) and separated via a 53-min gradient from 7 to 60% ACN in water with 0.1% formic acid at a constant flow of 300 nl/min. Afterward, the column was washed and reequilibrated. The eluting peptides were directly injected in the mass spectrometer and analyzed with a Top15 data-dependent acquisition method. After a full scan from 300 to 1650 *m/z* at resolution 60,000, the 15 most abundant precursor ions were fragmented with a normalized collision energy of 27, and the fragment spectra were recorded with a resolution of 30,000 and the precursors dynamically excluded for 20 s. Singly charged precursors and ions with unassigned charged state were excluded from fragmentation.

Peak extraction, peptide identification, and label-free quantification (LFQ) were performed with MaxQuant 1.6.5 with standard settings and activated LFQ.

In brief, extracted spectra were searched against the Human Swiss-Prot database (downloaded on 11.9.2017, including 20,205 forward sequences and supplemented with 246 laboratory contaminants by the software; decoy sequences were generated on the fly by MaxQuant). The theoretical digest with a maximum of two missed cleavage sites was performed with trypsin that cleaves peptide bonds mainly at the carboxyl group of lysine and arginine without proline inhibition. Carbamidomethylation of cysteine was applied as fixed modification, and oxidation of methionine as well as acetylation of the protein N terminus were defined as variable modification. For identification, the precursor ions need to pass a mass-tolerance threshold of 4.5 ppm and the associated fragment ions of 20 ppm. False discovery rate filtering was applied on peptide spectrum matches and on protein level, to limit false positives to 1%.

LFQ was applied by the LFQ algorithm implemented in MaxQuant using standard settings. Differentially abundant proteins were detected with Perseus 1.6.6 after transforming the LFQ values to log2 scale. For statistical analysis, only forward hits from the Swissprot database were taken into account that were quantified in all three replicates obtained from cells expressing GFP-USP11. For the GFP controls, imputation was performed to replace NaN values for the statistical analysis.

A two-sample, one-sided *t* test with an s0 of 1 and 5% false discovery rate correction was applied to identify proteins that were significantly enriched in the GFP-USP11 samples.

### C. elegans

According to standard procedures, *C. elegans* were maintained at 20 °C on nematode growth medium plates seeded with HB101 *E. coli*. RNAi was induced by feeding dsRNA as described previously (36). The knockdown efficiency was quantified by qPCR (37), analyzing mRNA levels of H34C03.2. Autophagic activity was investigated using age-synchronized nematodes that express GFP::LGG1 (ex[Plgg-1::GFP::lgg-1/pRF4], kind gift of Beth Levine) or GFP::LGG2 (VIG9 unc119(ed3)III; Is[unc-119(+); lgg-2p::gfp::lgg-2]) (38). Worms were cultivated under RNAi conditions at 20 °C and, at the first day of adulthood, were treated with dimethyl sulfoxide or bafilomycin A1 for 6 h (GFP::LGG1) or 2 h (GFP::LGG2), respectively. Thereafter, worms were lysed and GFP::LGG1/2 as well as tubulin protein levels were evaluated by immunoblotting, using NuPAGE 4 to 12% Bis-Tris gels (Invitrogen) for protein separation and antibodies directed against GFP (BioLegend, 902602) and tubulin (Sigma-Aldrich, T9026). Alternatively, these worms were imaged by confocal fluorescence microscopy using the laser scanning microscope LSM710 (Zeiss). For paralysis analysis, age-synchronized CL2006 (dvIs [Punc-54::hAß42/pRF4]) nematodes were cultivated at 15 °C on RNAi plates. Starting at the first day of adulthood, worms were transferred onto fresh RNAi plates daily and were tested for paralysis by tapping their nose with a platinum wire. Worms that moved their head but failed to move their body were scored as paralyzed. Dead nematodes or those that showed other phenotypes were not included into the statistics. Human Aβ42 levels were investigated by immunoblotting. CL2006 worms were lysed at day 1 or day 8 of adulthood, and proteins were detected using antibodies directed against Aβ42 (Biolegend, SIG-39329).

## Statistics

All statistical tests for experimental data were performed using GraphPad Prism; *p* values less than 0.05 were considered to be significant. Data were analyzed by *t* test or ANOVA, as appropriate. Statistical tests and n numbers are indicated in figure legends.

## Data availability

The mass spectrometry proteomics data have been deposited to the ProteomeXchange Consortium via the PRIDE (39) partner repository with the dataset identifier PXD022143.

## Supporting information

This article contains supporting information.

## Conflict of interest

The authors declare that they have no conflicts of interest with the contents of this article.
