# Supplementary material for: The deubiquitinase USP11 is a versatile and conserved regulator of autophagy
Source: J Biol Chem. 2021 Sep 30;297(5):101263. doi: 10.1016/j.jbc.2021.101263 (PMC8546420; doi:10.1016/j.jbc.2021.101263)
Supplement: Supplementary file 2 — Figures S1–S3 [file mmc2.pdf]

Supporting information

The deubiquitinase USP11 is a versatile and conserved regulator of autophagy

Mila Basic [1], Alexandra Hertel [1], Justyna Bajdzienko [1], Florian Bonn [1], Mariana Tellechea [2], Alexandra Stolz [2], Andreas Kern [3], Christian Behl [3] & Anja Bremm [1,*]

[1] Institute of Biochemistry II, Goethe University Frankfurt - Medical Faculty, University Hospital, Theodor-Stern-Kai 7, 60590 Frankfurt am Main, Germany
[2] Buchmann Institute for Molecular Life Sciences, Goethe University Frankfurt, Max-von-Laue-Str. 15, 60438 Frankfurt am Main, Germany
[3] Institute of Pathobiochemistry, University Medical Center of the Johannes Gutenberg University, Duesbergweg 6, 55128 Mainz, Germany

* Corresponding author: bremm@em.uni-frankfurt.de

Content:

- Supplemental figures S1-S3
- Supplemental table: USP11(C318S) interactome – protein identifications (provided as separate Excel file)

Figure S1

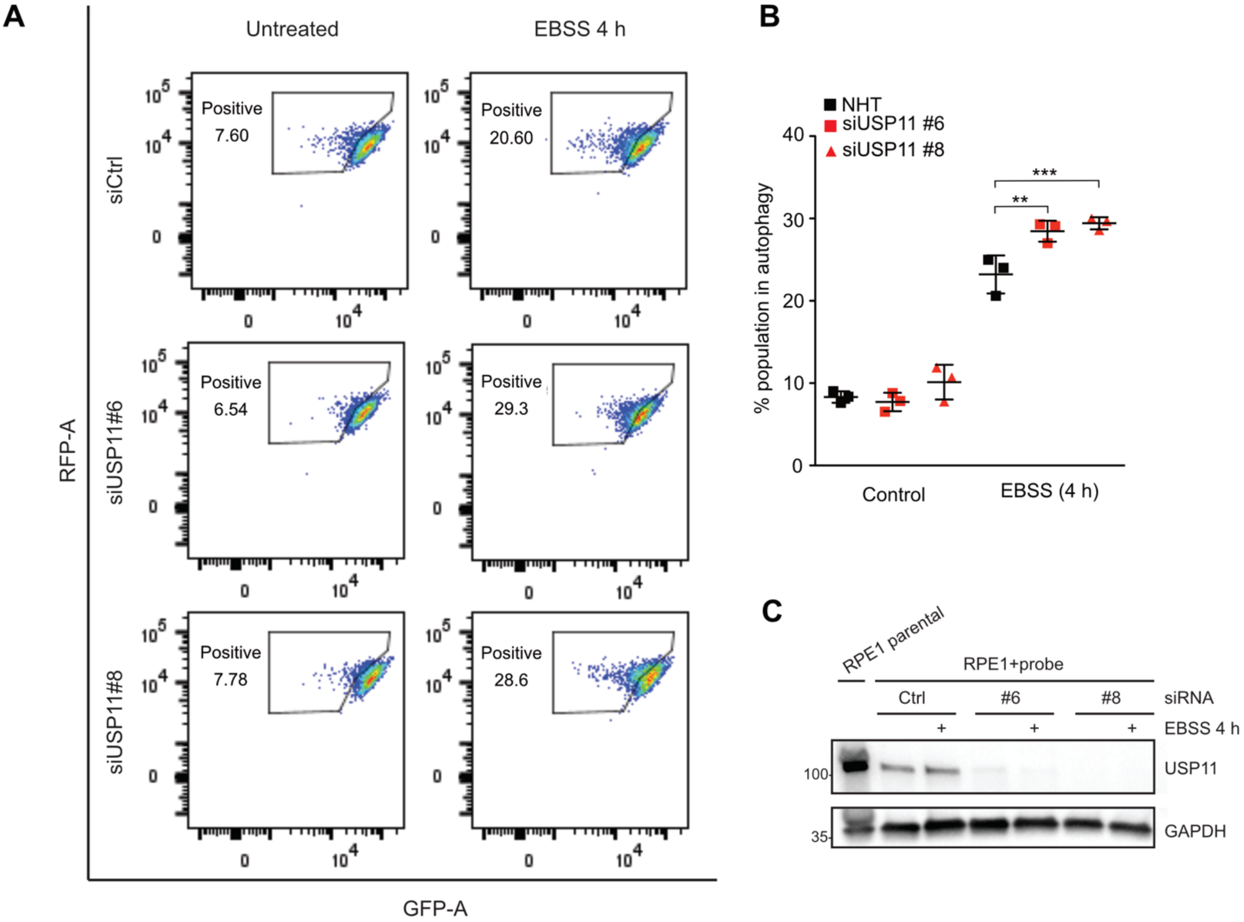

Supplemental Figure S1.

A Scatter plot showing USP11 knockdown via RNA interference with two different USP11-targeting sequences in RPE1 cells expressing the GFP-LC3-RFP autophagy probe decreased GFP/RFP ratios upon amino acid starvation (4 h EBSS treatment), indicative of higher autophagic flux.

B Quantification of autophagy-positive cells based on gates shown in (A) revealed a statistically significant increase in autophagy in both USP11 knockdown cell populations compared to siRNA control cells (p-value < 0.01 for siUSP11#6, and p-value <0.001 for siUSP11#8, 2way ANOVA, and Bonferroni posttest, N=3).

C Western blot control of knockdown efficiency confirmed successful downregulation of USP11 protein level.

Figure S2

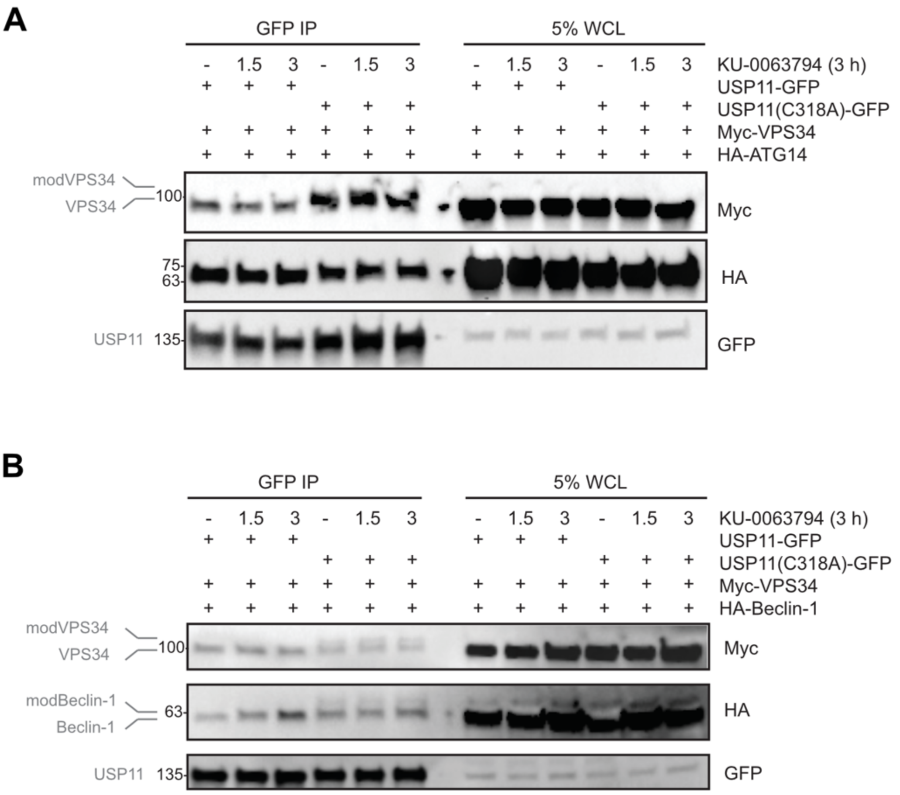

Supplemental Figure S2.

A Co-immunoprecipitation of USP11-, or USP11(C318S)-GFP with HA-ATG14 and Myc-VPS34 in 293 cells suggested a stable interaction of the three proteins, irrespective of autophagy induction, or activity of USP11.

B Similar to (A), USP11 co-precipitated the PI3KC3 complex component Beclin-1.

Figure S3

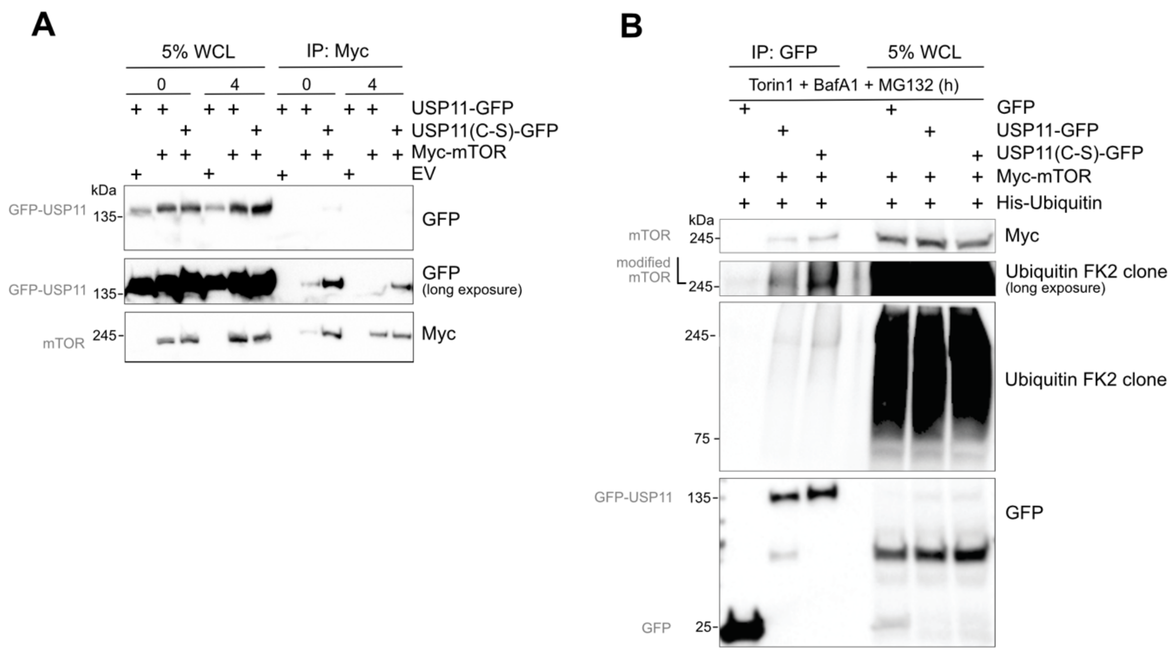

Supplemental Figure S3.

A Co-immunoprecipitation of USP11-GFP and myc-mTOR in control or Torin 1-treated 293 cells confirmed interaction of both proteins. Myc-mTOR co-precipitated more catalytic inactive USP11(C318S) as compared to the wildtype DUB.

B Co-immunoprecipitation of USP11-GFP and myc-mTOR in control or Torin 1-, Baf A1- and MG132-treated 293 cells expressing His-ubiquitin. Data suggests increased levels of ubiquitinated mTOR when co-precipitated with catalytic inactive USP11(C318S).
